# COVID-19 pandemic impact on mental health in children: a call for longitudinal datasets on prevalence of post-traumatic stress disorder

**DOI:** 10.1186/s43045-022-00266-1

**Published:** 2022-12-05

**Authors:** Gowda Parameshwara Prashanth

**Affiliations:** Department of Paediatrics, College of Medicine and Health Sciences, National University of Science and Technology, Muscat, Oman

## Commentary

Mental health in children is intricate with psychological, social, and physical environments acting as key factors influencing the health status and the opposing outcomes and hence difficult to forecast. Important contextual risk factors such as natural calamities, migration, political conflicts, and socioeconomic adversities could produce negative mental health outcomes in childhood. Recent medical literature is abundant with empirical studies reporting adverse mental health symptoms and health behaviors among children and adolescents during the COVID-19 pandemic [1]. In this regard, the paper by Karbasi and Eslami on the prevalence of post-traumatic stress disorder (PTSD) during the COVID-19 pandemic in children is remarkable [2].

Studies addressing the pandemic-related effects on mental health (“extended residuals”) by measuring psychological distress and well-being have shown that the initial outbreak of the COVID-19 pandemic led to a notable deterioration from existing mental health-related trajectories among young adults [3]. Analysis of pre- and mid-pandemic data suggested that preexisting health conditions increase the risk of poorer mental health outcomes apart from disorders such as stress, anxiety, depression, insomnia, and PTSD [2–4].

However, concerns have been raised regarding the relevance and validity of reported systematic reviews as well as the fact that most studies do not meet the PTSD exposure criterion among pediatric and adolescent populations [4, 5]. The heterogeneity of studies, the variety of instruments used to assess psychological distress, and a small number of studies measuring PTSD are highlighted recently [6]. Considering the methodology of review and the analysis of the testified data, similar criticisms are applicable to the current review by Karbasi and Eslami as well. This calls for a rigorous approach to assessing and reporting mental health outcomes such as PTSD.

It may also be pertinent to consider historical experiences from previous large-scale communicable disease outbreaks to address the persuasive issues threatening the validity of the results from current systematic reviews. During the severe acute respiratory syndrome (SARS) pandemic in 2003, the prevalence of PTSD in adult survivors was reported as high as 38.8% 1 year after recovery [7]. However, two decades later, following vigorous standards of a systematic review, Liu et al. (2021) reported that data is insufficient to permit reliable statistical analysis on PTSD following the SARS outbreak [8]. Similarly, limited parent-reported data during the H1N1 epidemic have indicated that up to 30% of children were experiencing post-traumatic stress following the epidemic [9]. There were no longitudinal studies published subsequently on this topic, and even warnings about major gaps in pediatric surge capacity in the event of an infectious disease pandemic remained unattended [10].

Among adults with COVID-19, the prevalence of PTSD as high as 96.2% during the hospital stay fell to 12.4% at the time of discharge, and after discharge, the severity of PTSD was aggravated [8, 11]. It is notable that among children, as reported in the current review, only 2 out of 10 studies were longitudinal. The importance of having further data from longitudinal studies with a clearly defined timeframe in the evaluation of PTSD cannot be overemphasized.

The aforementioned points of contention, therefore, make a strong case for reporting and corroboration of diagnostic screening for PTSD and follow-up to assess the “true burden” of PTSD among young children, adolescents, and adults. In addition to the use of *DSM-5* symptoms, the questions on direct vs indirect exposure and the eligibility of the pediatric population to meet the PTSD exposure criteria need to be addressed [5]. Data collected at multiple time points rather than cross-sectional would be more suitable to evaluate post-pandemic stress trajectories as well as the protective and risk factors involved.

It is remarkable that in studies concerning other disasters such as terrorism, floods, earthquake, and hurricane, self-reported measures are more frequent than other-reported measures [6]. Therefore, the authors of the current meta-analysis have correctly pointed out that children and adolescents may be more reliable as informants of self-experience compared to parents or others.

About half of the world’s children confronted school closures during the COVID-19 pandemic with a negative cascading effect of social isolation. The stress involved in online learning compounded by poor network connectivity, logistic issues, inadequate technical support, etc. brought about unprecedented fluctuations in their social life. With schools’ reopening subsequently, separation anxiety, stress, frustration, sadness, and struggle enhanced by fear of life, and uncertainty of the pandemic could result in behavioral dysregulation and affect the mental well-being of young children. On the other hand, data from adolescent cohorts (12 studies, *n* = 12,262) have shown that the pandemic had a significant impact on youth mental health and was mainly linked with depression and anxiety [12].

The present and past pandemic disasters have demonstrated that socioeconomic and environmental impacts of large-scale infection outbreaks and subsequent medical and public health responses may produce a stressful state of mental health that families and children find traumatic. Among the associated factors that could deepen the traumatic effect of the pandemic and influence outcomes such as PTSD, the issue of child maltreatment and psychological and physical abuse of the young remains at the forefront [13].

## Conclusions

There is overwhelming evidence suggesting that most people are resilient after a disaster [14]. Considering the extent and extraordinary nature of the pandemic, weighing the implications of PTSD on mental health needs a thorough relook. In view of the limited availability of high-quality data, perhaps it is premature to create hype around post-pandemic PTSD among young children. Early reactions and time to short-term recovery following a disaster like COVID-19 pandemic could provide important information about the psychological functioning of children and adolescents, with or without coronavirus infection, over time. In the meantime, validated instruments such as the Children and Adolescent Psychological Distress Scale (CAPDS-10) that measure perceived psychological distress over shorter time frames could be helpful for crisis assessment as well as to plan preventive strategies in pediatric and adolescent age groups [15].

Consequently, prompt identification of early pointers to psychological distress and provision of mental health services and clinical interventions to help remediate their symptoms need prioritization. As part of future pandemic preparedness, appropriate physician and parental educational activities need to be planned to prevent, detect, and treat early warning symptoms of PTSD.

## Data Availability

Not applicable

## Abbreviations

COVID19: Coronavirus disease 2019
PTSD: Post-traumatic stress disorder
CAPDS-10: Children and Adolescent Psychological Distress Scale
SARS: Severe acute respiratory syndrome
